# Hepatitis C virus impairs natural killer cell activity via viral serine protease NS3

**DOI:** 10.1371/journal.pone.0175793

**Published:** 2017-04-14

**Authors:** Chang Mo Yang, Joo Chun Yoon, Jeon Han Park, Jae Myun Lee

**Affiliations:** 1 Department of Microbiology and Immunology, Institute for Immunology and Immunological Diseases and Brain Korea 21 PLUS Project for Medical Sciences, Yonsei University College of Medicine, Seoul, Republic of Korea; 2 Department of Microbiology and Tissue Injury Defense Research Center, Ewha Womans University School of Medicine, Seoul, Republic of Korea; Saint Louis University, UNITED STATES

## Abstract

Hepatitis C virus (HCV) infection is characterized by a high frequency of chronic cases owing to the impairment of innate and adaptive immune responses. The modulation of natural killer (NK) cell functions by HCV leads to an impaired innate immune response. However, the underling mechanisms and roles of HCV proteins in this immune evasion are controversial, especially in the early phase of HCV infection. To investigate the role of HCV nonstructural proteins especially NS3 in the impairment of NK functions, NK cells were isolated from the PBMCs by negative selection. To assess the direct cytotoxicity and IFN-γ production capability of NK cells, co-cultured with uninfected, HCV-infected, HCV-NS3 DNA-transfected Huh-7.5, or HCV-NS replicon cells. To determine the effect of an NS3 serine protease inhibitor, HCV-infected Huh-7.5 cells were treated with BILN-2061. Then, NK cells were harvested and further co-cultured with K-562 target cells. NK cell functions were analyzed by flow cytometry and enzyme-linked immunosorbent assay. When co-cultured with HCV-infected Huh-7.5 cells, the natural cytotoxicity and IFN-γ production capability of NK cells were significantly reduced. NK cell functions were inhibited to similar levels upon co-culture with HCV-NS replicon cells, NS3-transfected cells, and HCV-infected Huh-7.5 cells. These reductions were restored by BILN-2061-treatment. Furthermore, BILN-2061-treatment significantly increased degranulation against K-562 target cells and IFN-γ productivity in NK cells. Consistent with these findings, the expression levels of activating NK cell receptors, such as NKp46 and NKp30, were also increased. In HCV-infected cells, the serine protease NS3 may play a role in the abrogation of NK cell functions in the early phase of infection through downregulation of NKp46 and NKp30 receptors on NK cells. Together, these results suggest that NS3 represents a novel drug target for the treatment of HCV infections.

## Introduction

Hepatitis C virus (HCV) is an enveloped, positive-sense RNA virus belonging to the *Flaviviridae* family [1]. Approximately 170 million people in the world are infected by HCV. HCV infection is characterized by its chronicity. About 80% of the HCV infected patients develop chronic hepatitis owing to impairment of the innate and adaptive immune responses. Chronic hepatitis progresses to liver fibrosis, cirrhosis, and hepatocellular carcinoma (HCC). Although impairment of the adaptive immune responses by HCV infection has been investigated previously, the mechanisms underlying the impairment of innate immune responses, especially the natural killer cell (NK) responses, are unclear [2, 3].

NK cells constitute a major component of the intrahepatic lymphocytes, and they mediate innate immune responses against several pathogens [4, 5]. NK cell function lies at the front line of defense against viral infections because NK cells recognize and rapidly kill virus-infected cells at the early phase of infection [4, 6, 7]. The outcomes of the engagement between NK cell receptors and target cell ligands are determined through the balance of signals from inhibitory and activating pathways. NK cell inhibitory receptors, such as NKG2A/CD94 or killer cell Ig-like receptors (KIR), recognize self or normal cells through the expression of class I major histocompatibility complex (MHC) molecules on target cells to prevent cytolysis. On the other hand, activating receptors, such as NKp46, NKp30, NKp44, and NKG2D, transduce activating signals upon binding to ligands on target cells whose class I MHC molecules are downregulated. NK cells directly lyse target cells through the secretion of the cytotoxic granules, granzyme and perforin [4, 8]. In addition, NK cells secrete proinflammatory cytokines such as interferon (IFN)-γ and tumor necrosis factor (TNF)-α [6, 9]. These cytokines exert a regulatory function on components of the adaptive immune system, including T cells, dendritic cells (DCs), and macrophages [6, 10].

It has been suggested that HCV alters the innate immune response at multiple levels. HCV-infected cells evade NK cell lysis at the early phase of infection. HCV activates regulatory T (Treg) cells, which secrete transforming growth factor (TGF)-β and interleukin (IL)-10 [11]. In our previous study, we reported that cell-to-cell contact with HCV-infected cells reduces the functional capacity of NK cells, and that the inhibition of NK cell function is associated with the downregulation of activating NK cell receptors [12]. These results indicate that a viral protein(s) may affect the infected cells, which in turn negatively affects NK cell functions.

The translation product of the HCV genome is a polyprotein that is cleaved by viral enzymes and host proteases to yield structural (S) proteins comprising Core, E1, E2, and non-structural (NS) proteins, including NS2, NS3, NS4A, NS4B, NS5A, and NS5B [2, 4]. Several HCV proteins have been proposed to contribute to the evasion of immune responses. The HCV Core protein upregulates MHC class I molecules on liver cells via p53 and TAP1, consequently impairing NK cell cytotoxicity [13]. HCV E2 protein, an envelope protein of HCV, may cross-link CD81 on NK cells, thereby decreasing the release of IFN-γ and cytotoxic granules [10, 14]. Furthermore, HCV NS3/4A can cleave the adaptor molecules, IPS-1 and TRIF [15], while HCV NS5A downregulates the expression of NKG2D on NK cells via TLR4, thereby impairing NK cell functions [16].

In this study, we attempted to identify the role of HCV NS3 protein that modulate NK cell functions and it’s mechanism by analyzing the cell-to-cell interaction of NK cells with HCV-infected Huh-7.5 cells. We found that cell-to-cell contact with HCV NS3-transfected cells reduced NK cell functions to a similar extent as in HCV-infected cells. Furthermore, these reductions were restored by treatment of HCV-infected Huh-7.5 cells with the NS3 serine protease inhibitor, BILN-2061, and this restoration correlated with the increased expression of the activating NK cell receptors, NKp46 and NKp30. These findings suggest that the HCV serine protease NS3 plays a role in the impairment of NK cell functions in the early phase of infection.

## Materials and methods

### Cell lines

Human hepatoma Huh-7.5 cells (provided by C. Rice, Rockefeller University, New York, NY) were maintained in Dulbecco’s Modified Eagle Medium (DMEM) supplemented with 10% fetal bovine serum (FBS) and 1% penicillin/streptomycin (complete DMEM; all from HyClone, South Logan, UT). Human myelogenous leukemia K-562 cells (ATCC Number: CCL-243) were maintained in Roswell Park Memorial Institute (RPMI) 1640 medium supplemented with 10% FBS and 1% penicillin/streptomycin, and 2.05 mM l-glutamine (complete RPMI 1640; all from HyClone). HCV-NS replicon cells (provided by S. K. Jang, Pohang University of Science and Technology, Pohang, South Korea) were maintained in complete DMEM containing 500 μg/mL G418 (Duchefa Biochemie, Haarlem, Nederland) [17]. All cells were cultured at 37°C in a 5% CO_2_ incubator.

### Generation of HCVcc

HCVcc (genotype 2a, JFH-1 strain, plasmid provided by T. Wakita, National Institute of Infectious Diseases and Toray Industries, Tokyo, Japan) was produced as described previously [12, 18] and also HCV titer was determined as described previous studies [12, 19].

### Peripheral Blood Mononuclear Cell (PBMC) isolation and NK cell purification

PBMCs were isolated from the whole blood of healthy donors by using Ficoll-Paque (GE Healthcare Life Science, Piscataway, NJ) and then NK cells were isolated from PBMCs as described previously [12]. All donors provided written informed consent for the use of their blood, and the protocols were approved by the Institutional Review Board (IRB) of Severance Hospital Yonsei University Health System.

The purity of the NK cells was measured by LSR II flow cytometer (BD Biosciences, San Jose, CA) after staining the cells with anti-CD3-allophycocyanin (APC)-H7 and anti-CD56-PerCP-Cy5.5 or anti-CD56-APC antibodies (all antibodies were diluted 1:100 in FACS buffer) (BD Bioscience). The isolated NK cells checked frequency and used for following experiments.

### Degranulation of NK cells

Huh-7.5 cells (1 × 10^4^) were seeded in 96-well flat-bottom culture plates (Nunc, Roskilde, Denmark). After 24 h, Huh-7.5 cells were infected with HCVcc at a multiplicity of infection (MOI) of 1. After 72 h later, NK cells in complete RPMI 1640 were added to the uninfected, HCV-infected, HCV-NS3 DNA-transfected Huh-7.5, or HCV-NS replicon cells at a 1:1 ratio for 18 h. To determine the effects of NS3, NK cells were also co-cultured with HCV-NS3 DNA (provided by Eui-Cheol Shin, Korea Advanced Institute of Science and Technology, Daejeon, South Korea)-transfected Huh-7.5 cells or HCV-NS replicon cells. To evaluate the effect of an NS3 serine protease inhibitor, NK cells were co-cultured with BILN-2061-treated HCV-infected Huh-7.5 cells. Confirmed the direct cytotoxicity of NK cells as described previous study [12]. Then, NK cells were stained with anti-CD56-PerCP-Cy5.5 or anti-CD56-APC antibody, fixed with 1% formaldehyde, and analyzed with a flow cytometer (BD Biosciences) and the FlowJo_V10 software (Tree Star, Ashland, OR).

### IFN-γ production of NK cells

NK cells were co-cultured with the cells described above for 18 h, then harvested to 96-well round-bottom culture plates (Corning Inc.). To measure the intracellular IFN-γ levels, NK cells were further co-cultured with K-562 target cells then fixed and intracellular staining as described previous study [12]. Secreted IFN-γ in the collected supernatant was assessed by a human IFN-γ enzyme-linked immunosorbent assay kit (Enzo Life Sciences, Farmingdale, NY or ATGen, Seongnam, South Korea) according to the manufacturer’s instructions.

### Receptor expression of NK cells

To determine changes in the expression levels of NK cell receptors induced by HCV-infected cells, NK cells were co-cultured with the cells described above, and then stained with anti-NKp46-PE, anti-NKp30-PE, anti-NKG2D-APC (BD Biosciences), and anti-2B4-APC (BioLegend, San Diego, CA or BD Biosciences). Stained cells were analyzed with a flow cytometer.

### Confocal microscopy

For NS3 immunostaining, cells were seeded in four-well chamber slides (Nunc), washed with phosphate-buffered saline (PBS) (Hyclone), and fixed with 3.7% formaldehyde for 10 min at room temperature. Cells were permeabilized with 0.1% Triton X-100 in PBS buffer for 5 min at room temperature, and then blocked with 1% bovine serum albumin (BSA; Affymetrix, Cleveland, OH) in PBS buffer for 20 min at room temperature. After washing with PBS, cells were incubated with mouse monoclonal anti-HCV-Core antibody (Thermo Scientific, Grand Island, NY) diluted 1:300 in PBS or anti-HCV-NS3 antibody (Thermo Scientific) diluted 1:50 in PBS for 1 h at room temperature. Slides were washed with PBS and mounted using VECTASHIELD mounting medium with DAPI (Vector Laboratories, Burlingame, CA). Images were visualized on an LSM-700 confocal microscope (Carl Zeiss, Jena, Germany).

### Western blot analysis

Cells were lysed with RIPA buffer (50 mM Tris-Cl, pH 7.5, 150 mM NaCl, 1% NP-40, 0.5% sodium deoxycholate, and 0.1% SDS). Then, bicinchoninic acid assay was performed to determine the protein concentration. Cell lysates were separated on a 10% glycine/sodium dodecyl sulfate polyacrylamide gel by electrophoresis. Proteins were then transferred to a 0.45-μm nitrocellulose membrane (Bio-Rad, Hercules, CA). The membrane was blocked for 1 h with 5% skim milk in PBST buffer, and then incubated at 4°C overnight with anti-β-actin (Sigma-Aldrich, St. Louis, MO; 1:8,000), anti-HCV-NS3 (1:50), and anti-HCV-Core (1:1,000) in 3% BSA in PBST buffer. Horseradish peroxidase-conjugated anti-mouse IgG was used as the secondary antibody (1:5,000 or 1:8,000), and bands were visualized by electrochemiluminescence (Advansta, Menlo Park, CA) according to the manufacturer’s instructions.

### Statistical analysis

Student’s *t* tests and repeated-measures one-way analysis of variance were performed using GraphPad Prism 6 (GraphPad Software, San Diego, CA). *p*-Values of <0.05 were considered significant.

## Results

### HCV-infected Huh-7.5 cells and HCV NS replicon cells reduce the functional capacity of NK cells

To investigate the modulatory effect of HCV-infected Huh-7.5 cells on NK cell functions, NK cells were co-cultured for 18 h with HCV-infected Huh-7.5 cells. Then, NK cell cytotoxicity against K-562 cells was assessed by stained the expression of CD107a, a marker of NK cell cytotoxic granules. Then, IFN-γ productivity was measured by intracellular flow cytometry. Co-cultivation of NK cells with HCV-infected Huh-7.5 cells significantly reduced CD107a expression against K-562 cells and IFN-γ production compared with NK cells alone or NK cells co-cultured with uninfected Huh-7.5 cells (S1 Fig). These observations are in accordance with our previous data, where direct cell-to-cell contact NK cells and HCV-infected Huh-7.5 cells reduced the functions of NK cells [12].

**Fig 1:**
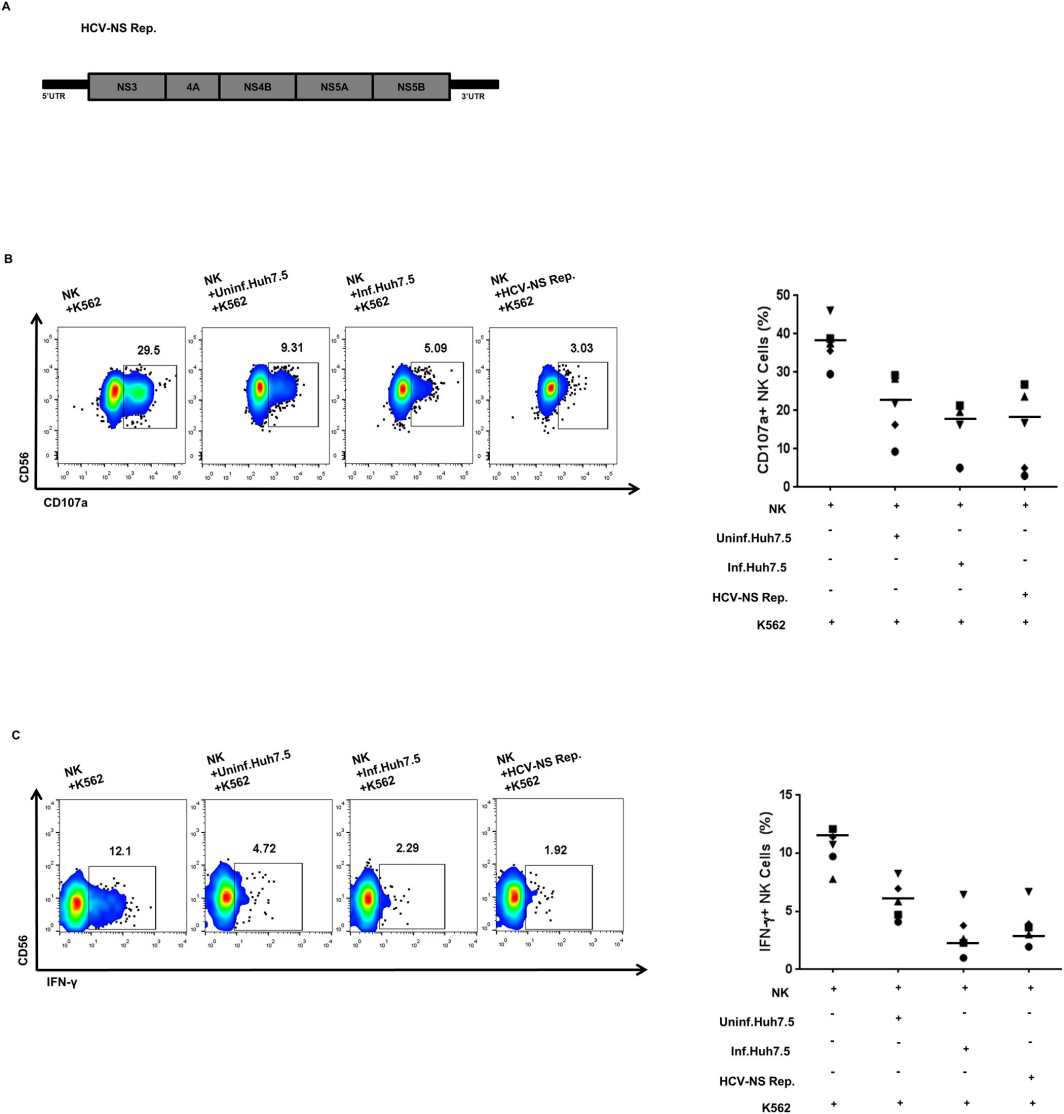
Hepatitis C Virus-Non-Structural (HCV-NS) protein-expressing cells reduce Natural Killer (NK) cell cytotoxicity and Interferon (IFN)-γ production. (A) Schematic diagrams of the HCV–NS replicon constructs. (B) Degranulation of NK cells after co-cultivation with HCV-infected Huh-7.5 cells or HCV–NS replicon cells. NK cells were pre-incubated with HCV-infected Huh-7.5 cells or HCV-NS replicon cells for 18 h, and harvested the NK cells then co-cultured with K-562 cells at a 1:1 ratio for 4 h. NK cell degranulation was measured by estimating CD107a expression. (C) IFN-γ production by NK cells after co-cultivation with HCV-infected Huh-7.5 cells or HCV–NS replicon cells. NK cells were pre-incubated with HCV-infected Huh-7.5 cells or HCV-NS replicon cells for 18 h, and harvested the NK cells then co-cultured with K-562 cells at a 1:1 ratio with treatment of 10 ng/mL recombinant human interleukin (IL)-12 and 100 ng/mL IL-15 for 6 h. IFN-γ production was assessed by intracellular staining of IFN-γ followed by flow cytometry. (B-C) Representative pseudo color plots obtained for five independent individuals. Bar presents the median value.

Next, to investigate whether the non-structural proteins of HCV can reduce NK cell functions, we co-cultured NK cells with HCV-NS replicon cells (Fig 1A). NK cytotoxicity and IFN-γ productivity were reduced by co-cultivation of NK cells with HCV-NS replicon cells, similar to the co-cultivation of NK cells with HCV-infected Huh-7.5 cells (Fig 1B and 1C). Together, these data demonstrate that HCV-infected cells regulate NK cell functions via cell-to-cell interaction and that HCV-NS proteins might be involved in this modulation.

### HCV-NS3 reduces the functional capacity of NK cells

**Fig 2:**
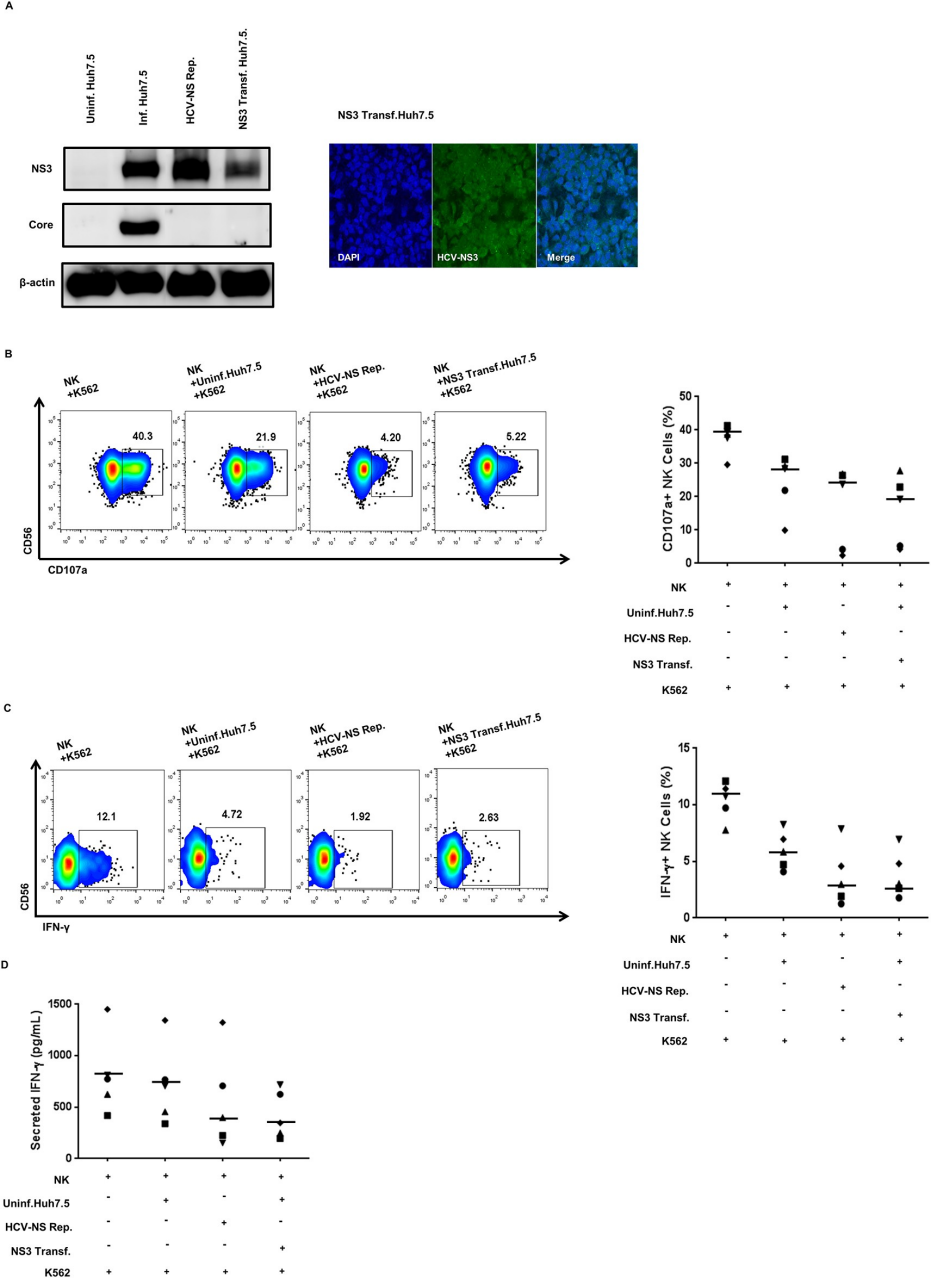
HCV-NS replicon and HCV-NS3-transfected Huh-7.5 cells attenuate NK cell functions. (A) Expression levels of HCV NS3 protein in HCV-NS replicon cells and HCV-NS3-transfected Huh-7.5 cells. Western blotting (*left panel*) was performed using anti-HCV-NS3 and anti-HCV-Core antibodies. As a loading control, human anti-β-actin was used. Confocal microscopy (*right panel*) was performed after 48 h of NS3 transfection. Transfected Huh-7.5 cells were fixed and stained with HCV-NS3 antibody (*green*) and 4′,6-diamidino-2-phenylindole (DAPI; *blue*). Transient transfection was carried out using Lipofectamine 2000 (Invitrogen) (B) NK cell degranulation after co-cultivation with NS3-transfected Huh-7.5 cells. NK cells were pre-incubated with uninfected cells, HCV-NS replicon cells, or HCV-NS3-transfected Huh-7.5 cells for 18 h, and the harvested NK cells were then co-cultured with K-562 cells at a 1:1 ratio for 4 h. NK cell degranulation was measured by estimating CD107a expression. (C) IFN-γ production by NK cells after co-cultivation with NS3-transfected Huh-7.5 cells. NK cells were pre-incubated with uninfected, HCV-NS replicon, or HCV-NS3-transfected Huh-7.5 cells for 18 h, and harvested the NK cells then co-cultured with K-562 cells at a 1:1 ratio with treatment of 10 ng/mL IL-12 and 100 ng/mL IL-15 for 6 h. IFN-γ production was assessed by intracellular staining of IFN-γ. (D) IFN-γ secretion by NK cells after co-cultivation with NS3 transfected Huh-7.5 cells. NK cells were pre-incubated with HCV-NS replicon or NS3-transfected Huh-7.5 cells for 18 h, and harvested the NK cells then co-cultured with K-562 cells at a 1:1 ratio with treatment of 10 ng/mL recombinant human IL-12 and 100 ng/mL IL-15 for 18 h. Secreted IFN-γ in the supernatant was measured by enzyme-linked immunosorbent assay (ELISA). (B-C) Representative pseudo color plots obtained for five independent individuals. (D) Data from five independent individuals. Bar presents the median value.

To investigate which HCV NS protein is responsible for the reduced NK cell cytotoxicity and IFN-γ production, we focused on NS3, because it acts as a serine protease and helicase and is therefore essential for viral replication [2]. To demonstrate the effect of NS3 on NK cell functions, we used the HCV-NS3 overexpression system. To verify NS3 expression, western blot analysis and confocal microscopy were performed in HCV-NS replicon cells and NS3-transfected Huh-7.5 cells. The expression level of NS3 in these two cell lines was comparable to that in HCV-infected Huh-7.5 cells (Fig 2A). The cytotoxicity and IFN-γ-production capability of NK cells was also reduced by co-cultivation with NS3-transfected Huh-7.5 cells, as in the case of HCV-NS replicon cells (Fig 2B and 2C). Reduced IFN-γ production by HCV-NS replicon cells and NS3-transfected Huh-7.5 cells was confirmed using ELISA (Fig 2D). These results indicate that NS3 expressed in the HCV-infected cells might play a role in the modulation of NK cell functions.

### Treatment of HCV-infected cells with an NS3 inhibitor, BILN-2061, restores NK cell functions

**Fig 3:**
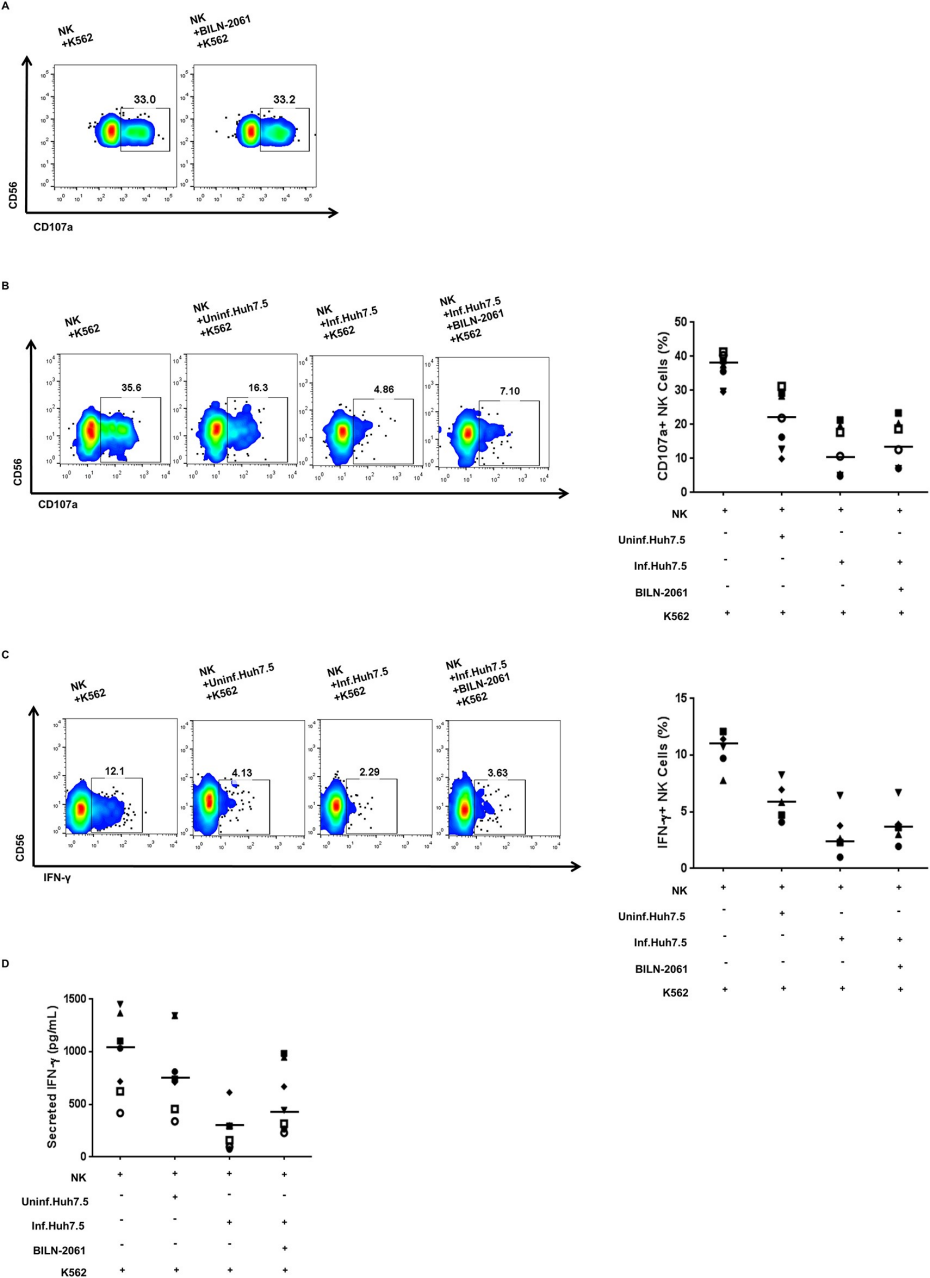
NK cell functions were restored after interaction with BILN-2061-treated HCV-infected Huh-7.5 cells. (A) NK cell degranulation was measured as described above. NK cells were seeded in a 96-well round bottom culture plate and treated with 400 nM of BILN-2061 for 18 h and then co-cultivation with K-562 cells at a 1:1 ratio for 4 h. (B) NK cell degranulation after co-cultivation with BILN-2061-treated HCV-infected Huh-7.5 cells. NK cells were pre-incubated with uninfected, HCV-infected, or BILN-2061-treated HCV-infected Huh-7.5 cells for 18 h, and harvested the NK cells then co-cultured with K-562 cells at a 1:1 ratio for 4 h. NK cell degranulation was measured by estimating CD107a expression. (C) IFN-γ production by NK cells after co-cultivation with BILN-2061-treated HCV-infected Huh-7.5 cells. NK cells were pre-incubated with uninfected, HCV-infected, or BILN-2061-treated HCV-infected Huh-7.5 cells for 18 h, and harvested the NK cells then co-cultured with K-562 cells at a 1:1 ratio with treatment of 10 ng/mL IL-12 and 100 ng/mL IL-15 for 6 h. IFN-γ production was assessed by intracellular staining of IFN-γ. (D) IFN-γ secretion by NK cells after co-cultivation with BILN-2061-treated HCV-infected Huh-7.5 cells. NK cells were pre-incubated with uninfected, HCV-infected, or BILN-2061-treated HCV-infected Huh-7.5 cells for 18 h, and harvested the NK cells then co-cultured with K-562 cells at a 1:1 ratio with treatment of 10 ng/mL IL-12 and 100 ng/mL IL-15 for 18 h. Secreted IFN-γ in the supernatant was measured by ELISA. (A-C) Representative pseudo color plots obtained for three, seven and five independent individuals. (D) Data from seven independent individuals. Bar presents the median value.

To verify the role of NS3 in the reduced NK cell degranulation and IFN-γ production, HCV-infected Huh-7.5 cells were treated with an NS3 inhibitor, BILN-2061. BILN-2061 (up to 700 nM) did not affect the cell viability of Huh-7.5 and Huh-7 cells (S2A Fig). BILN-2061-treatment decreased the expression levels of HCV Core protein and NS3 protein in a dose-dependent manner (S2B–S2D Fig). We determined the effects of BILN-2061 treatment on NK cells. Treatment with 400 nM of BILN-2061 did not affect NK cell degranulation directly (Fig 3A). After 4 h of HCV infection, Huh-7.5 cells were transferred to fresh medium containing 400 nM BILN-2061. Treatment of HCV-infected Huh-7.5 cells with BILN-2061 restored NK cells degranulation (from 4.86% in the untreated group to 7.10% in the treated group; Fig 3B). This restoration of function by BILN-2061-treatment was also observed in the case of IFN-γ production (Fig 3C) and secretion (Fig 3D). These observations corroborate the above data (Fig 2), connoting that NS3 plays a role in the modulation of NK cell functions.

### Restoration of NK cell functions upon BILN-2061-treatment is associated with increased NKp46 and NKp30 expression

**Fig 4:**
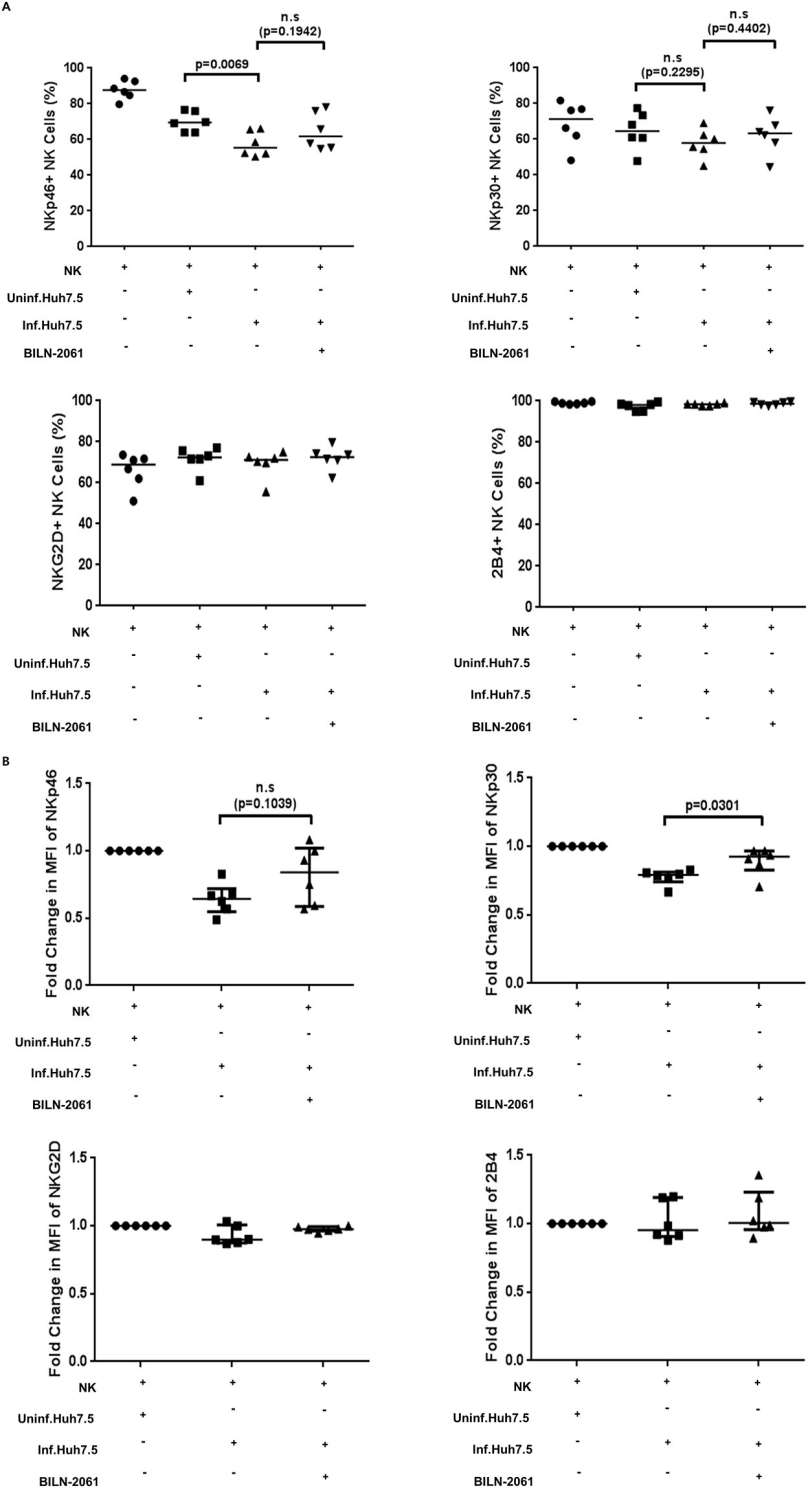
Activating NK cell receptors expression after interaction with BILN-2061-treated HCV-infected Huh-7.5 cells. (A) NK cells were co-cultured with uninfected, HCV-infected, or BILN-2061-treated HCV-infected Huh-7.5 cells for 18 h, and the frequency of expressing activating NK cell receptors was measured by flow cytometry. Frequencies of NKp46^+^, NKp30^+^, NKG2D^+^ and 2B4^+^ NK cells are shown. (B) Effect of BILN-2061-treated HCV-infected Huh-7.5 cells on the expression levels of activating receptors on NK cells. NK cells were co-cultured with uninfected, HCV-infected, or BILN-2061-treated HCV-infected Huh-7.5 cells for 18 h. Fold change in the mean fluorescence intensity (MFI) of NKp46, NKp30, NKG2D, and 2B4 on NK cells is shown as relative values to uninfected Huh-7.5 cells. Error bars indicate the median with interquartile range.

In our previous study, we reported that activating receptors expressed on NK cells surface are downregulated after co-cultivation with HCV-infected cells, and that this downregulation is correlated with the functional impairment of NK cells [12]. To investigate the mechanism of this functional impairment by HCV-infected cells, we examined the surface expression of various activating receptors after co-culture of NK cells with HCV-infected Huh7.5 cells and HCV replicon cells. In accordance with our previous report [12], co-culture of NK cells with HCV-infected cells decreased the NK cell population expressing activating receptors such as NKp46 and NKp30 (Fig 4A).

To investigate whether BILN-2061 affects the surface expression of NK cell activating receptors, we evaluated the surface expression of the activating receptors after treatment of HCV-infected Huh-7.5 cells with BILN-2061. Treatment of these cells with BILN-2061 increased not only the NK cell population expressing activating receptors (Fig 4A) but also the expression levels of the activating receptors (Fig 4B). Flow cytometric data showed that the NK cell population expressing NKp46 and NKp30 was decreased after co-culture with HCV-infected Huh-7.5 cells (Fig 4A). In contrast, these effects were not observed for NKG2D and 2B4 (Fig 4A). Previous study has indicated that NKp30 surface expression is downregulated (determined by assessing the mean fluorescence intensity [MFI]) following co-culture with HCV-infected Huh-7.5 cells [20]. We observed that the NKp46 and NKp30 expression levels of total NK cells were decreased after co-culture with HCV-infected Huh-7.5 cells. The surface expression levels were restored when NK cells were co-cultured with BILN-2061-treated HCV-infected Huh-7.5 cells. The fold change in MFI of NKp46 and NKp30 expression on NK cells increased upon co-culture with BILN-2061-treated HCV-infected Huh-7.5 cells (*p* = 0.1039 for NKp46 and *p* = 0.0301 for NKp30, Student’s *t* test; n = 6, respectively; Fig 4B).

## Discussion

Approximately 80% of untreated HCV-infected patients develop chronic hepatitis, and therefore, HCV infection represents a considerable public health burden [21]. This chronicity has been attributed to insufficient development of HCV-specific cytotoxic T lymphocytes owing to impairment of the innate immune response against the early phase of HCV infection [2, 22, 23].

The evading mechanisms adopted by HCV to protect the virus from innate immune responses, notably NK cells, in early phase of infection have not been clarified. The importance of NK cells in anti-viral immune responses has prompted studies on the interactions between NK cells and HCV, and several studies have suggested that NK cells play a role in the clearance of HCV. Genetic studies have demonstrated that genes encoding the inhibitory NK cell receptor KIR2DL3 and its human leukocyte antigen C group 1 (HLA-C1) ligand directly influence resolution of HCV infection [24, 25]. On the contrary, some studies have reported that NK cell dysfunction is associated with chronic HCV infection. Increased NKG2A expression is a consistent finding in chronic HCV, suggesting the inhibition of NK cell functions [26–28]. Moreover, conflicting results have been reported for NCR expression levels in chronic HCV [28–31]. Our previous findings suggest that the impairment of NK cell functions as a consequence of cell-to-cell interaction among NK cells and HCV-infected Huh-7 and/or Huh-7.5 cells contribute the chronicity of HCV infection [12]. In this study, using HCV-NS replicon cells and an NS3 overexpression system, we demonstrated that viral NS proteins could inhibit NK cell functions (Figs 1 and 2). We also investigated the changes in the surface expression of activating receptors on NK cells upon cell-to-cell interaction with HCV-infected Huh-7.5 cells (Fig 4).

In this study, we used human hepatoma cell line Huh-7.5 cells which is one of the well-known cell lines for highly permissive to infectious HCV. There are many available human hepatoma cell lines, for example, Huh-7, Huh-7.5, Hep3B, HepG2, PLC/PRF/5 and etc. However, HCV infection *in vitro*, just Huh-7 and Huh-7.5 cells are highly permissive for infectious HCV. Therefore, many researchers who use HCVcc system *in vitro* generally use Huh-7 and/or Huh-7.5 cell lines [11, 20, 21, 32, 33]. A recent study showed possibility that Hep3B and PLC cells are also permissive for HCV infection, but these cells have a significantly less level of infectious susceptibility than Huh-7 and Huh-7.5 cells [34].

In addition to the limitation of this study, we have to considered that tumor cells mainly downregulate NK cell activating receptors or upregulate NK cell inhibitory receptors [35,36]. These alterations induced an impairment of NK cell activity. In previous studies also found that HCV-uninfected Huh-7 or Huh-7.5 cells inhibit the NK cell functions [12, 20]. Likewise, we confirmed that NK cell functional capacity reduced by interaction with HCV-uninfected Huh-7.5 cells.

In accordance with our previous report [12], we observed that co-cultivation of NK cells with HCV-infected Huh-7.5 cells significantly reduced the expression of CD107a against K-562 cells, as well as IFN-γ production. We also observed that NK cell cytotoxicity and IFN-γ productivity was reduced upon co-cultivation of NK cells with HCV-NS replicon cells expressing HCV NS proteins. These observations indicate that HCV-infected cells regulate NK cell functions by cell-to-cell interaction and that HCV NS proteins might be involved in this modulation.

To investigate which HCV NS protein is responsible for the reduced NK cell cytotoxicity and IFN-γ production, we focused on NS3, because NS3/4A have a major key role in the immune evasion of HCV. When NS3/4A is overexpressed, it cleaves the adaptor molecules IPS-1 and TRIF, of RIG-I and TLR3, respectively [2, 15, 37], thereby blocking RIG-I and TLR-3 signaling [2]. These results suggest that HCV NS3/4A plays a crucial role in the evasion mechanism against host innate immune responses. Additionally, the NS3 serine protease cleaves the polyprotein of HCV to generate individual NS proteins. Hence, it is important to understand the role of HCV NS3 protease in the evasion mechanism against innate immune responses, especially NK cells. NK cells functional capacity (cytotoxicity and IFN-γ production) was greatly reduced upon co-cultivation with NS3-transfected Huh-7.5 cells, as well as with HCV-NS replicon cells, suggesting that NS3 expressed in the HCV-infected cells might play a role in the modulation of NK cell functions.

To verify the role of NS3 in the reduced NK cell degranulation and IFN-γ production, HCV-infected Huh-7.5 cells were treated with an NS3 inhibitor, BILN-2061. Treatment of HCV-infected Huh-7.5 cells with BILN-2061 restored NK cell degranulation and IFN-γ production, corroborating the hypothesis that HCV-NS3 plays a crucial role in the evasion mechanism against NK cell-mediated innate immune response. We also observed that the functions of NKp46^+^ and NKp30^+^ NK cells were restored after interaction with HCV-infected Huh-7.5 cells treated with BILN-2061. Thus, HCV-NS3 reduces NK cell anti-viral functions by downregulating the expression of activating receptors such as NKp46 and NKp30 on NK cells. Therefore, NS3 might represent a promising drug target, and NS3 inhibitors could be used to recover NK cell functions during HCV infection.

BILN-2061 is a non-covalent competitive and macro-cyclic β-stranded inhibitor against HCV genotype 1 and 2 [38–40]. HCV NS3 serine protease contains a classical catalytic triad, and BILN-2061 specifically acts against this active site [38, 40]. BILN-2061-treatment restored functional impairment in NK cells co-cultivated with HCV-infected cells. To confirm these findings, further investigations using HCV NS3 mutants need to be conducted. NK cell dysfunction due to direct interaction between HCV-infected cells and NK cells may be explained by several mechanisms. First, HCV-infected cells also express HLA-E, which is a ligand for NKG2A/CD94, inhibitory receptors on NK cells [20, 41]. However, the results of our and other previous studies found that HLA-E expression on HCV-infected cells did not increased [12, 20]. Furthermore, previous studies have demonstrated that a direct antagonistic interaction between HCMV protein pp65 and NKp30 activating receptor reduces NK cell cytotoxicity through dissociation of the linked CD3 ζ-chain adaptor protein from NKp30 [20, 42]. NKp46, NKp30, and CD16 are associated with the same ζ-chain adaptor molecule [43] and share the same signaling pathway [44, 45]. In a previous study, *ex vivo* NK cells from HIV-infected individuals showed reduced CD16 expression, leading to impaired NK cell functions through reduced NKp46 expression [45]. It is also possible that unknown antagonistic NKp46 and NKp30 ligands induced on HCV-infected cells inhibit NK cell functions. Unfortunately, many of the activating and inhibitory ligands for NKp46 and NKp30 remain uncharacterized. Therefore, we could not clarify the evasion mechanism of HCV against NK cell responses in terms of the receptor-ligand interaction. We hope that our study prompts further investigations on the ligands of the NKp46 and NKp30 receptors in HCV infection.

Another study has demonstrated that NS5A of HCV binds to TLR4 on monocytes, and induces IL-10 and TGF-β production, while inhibiting IL-12 production, which downregulates NKG2D on NK cell surfaces and impairs NK cell functions [16, 46]. These observations indicate that immunosuppressive cytokines might play an critical role in NK cell dysfunction at the early phase of HCV infection. HCV-NS3 protease might inhibit NK cell functions indirectly by modulating the expression of surface molecules on HCV-infected cells. We will further investigate to reveal detailed mechanisms of NS3-mediated suppression of NK cell functions.

In summary, this study revealed that NK cell functions are significantly impaired in the early phase of HCV infection *in vitro*, and that this effect might be mediated via viral serine protease and downregulation of the activating receptors, NKp46 and NKp30. Further studies investigating the signaling processes stimulated by NS3 may help understand the detailed evading mechanisms of HCV against innate immune responses, especially NK cells. Together, our results suggest that NS3 might represent a novel drug target for HCV infection, and that inhibition of NS3 could help rescue immune responses against HCV infection.

## Supporting information

S1 Fig Reduced NK cell cytotoxicity and IFN-γ production after direct contacted with HCV-infected Huh-7.5 cells. (A) Expression of HCV-Core protein in HCV-infected Huh-7.5 cells. Huh-7.5 cells were infected with HCV-JFH1 (MOI = 1), and three days later, cells were fixed and stained for HCV Core protein (*green*) immunofluorescence with DAPI (*blue*) nuclear staining. (B) NK cell degranulation after co-cultivation with HCV-infected Huh-7.5 cells was measured as described in Fig 1. (C) IFN-γ production by NK cells after co-cultivation with HCV-infected Huh-7.5 cells. IFN-γ production was assessed by intracellular staining of IFN-γ as described in Fig 1. (B) Representative pseudo color plots obtained for seven independent individuals. (C) Representative pseudo color plots obtained for five independent individuals. Bar presents the median value.
(TIF)

S2 Fig Determination of the optimal concentration of BILN-2061, a serine protease that cleaves the HCV-NS3 inhibitor. (A) Effect of BILN-2061 on cell viability. Huh-7.5 and Huh-7 cells were seeded in a 96-well flat bottom culture plate and infected with HCV at an MOI of 1 in Huh-7.5 cells and at an MOI of 10 in Huh-7 cells. After 4 h, the supernatant was removed, cells were transferred to complete DMEM, and treated with BILN-2061 (100–700 nM) for 48 h. Cell viability assay was performed using the CCK-8 kit (Dojindo Molecular Technologies, Japan). BILN-2061 did not affect cell viability up to 700 nM. (B-C) Effect of BILN-2061 on HCV replication. HCV-infected Huh-7.5 cells were treated with BILN-2061 (100–400 nM) for 48 h. HCV replication was determined by estimating HCV-Core expression levels by using flow cytometry (stained with anti-HCV-Core antibody). HCV replication in HCV-infected Huh-7.5 cells was reduced by BILN-2061-treatment in a dose-dependent manner. Representative pseudo color plots of the results from three independent experiments, and their bar graphs (C). (D) Effect of BILN-2061 on NS3 protein expression in HCV-infected Huh-7.5 cells. HCV-infected Huh-7.5 cells were treated with BILN-2061 (100–400 nM) for 48 h. Western blotting was performed using anti-HCV-NS3 and anti-β-actin antibodies. BILN-2061-treated HCV-infected Huh-7.5 cells reduced NS3 expression in a dose-dependent manner. Thus, the optimal concentration was determined to be 400 nM BILN-2061 for further experiments.
(TIF)
