# Supplementary figures and images for: Hepatitis C virus impairs natural killer cell activity via viral serine protease NS3
Source: PLoS One. 2017 Apr 14;12(4):e0175793. doi: 10.1371/journal.pone.0175793 (PMC5391949; doi:10.1371/journal.pone.0175793)

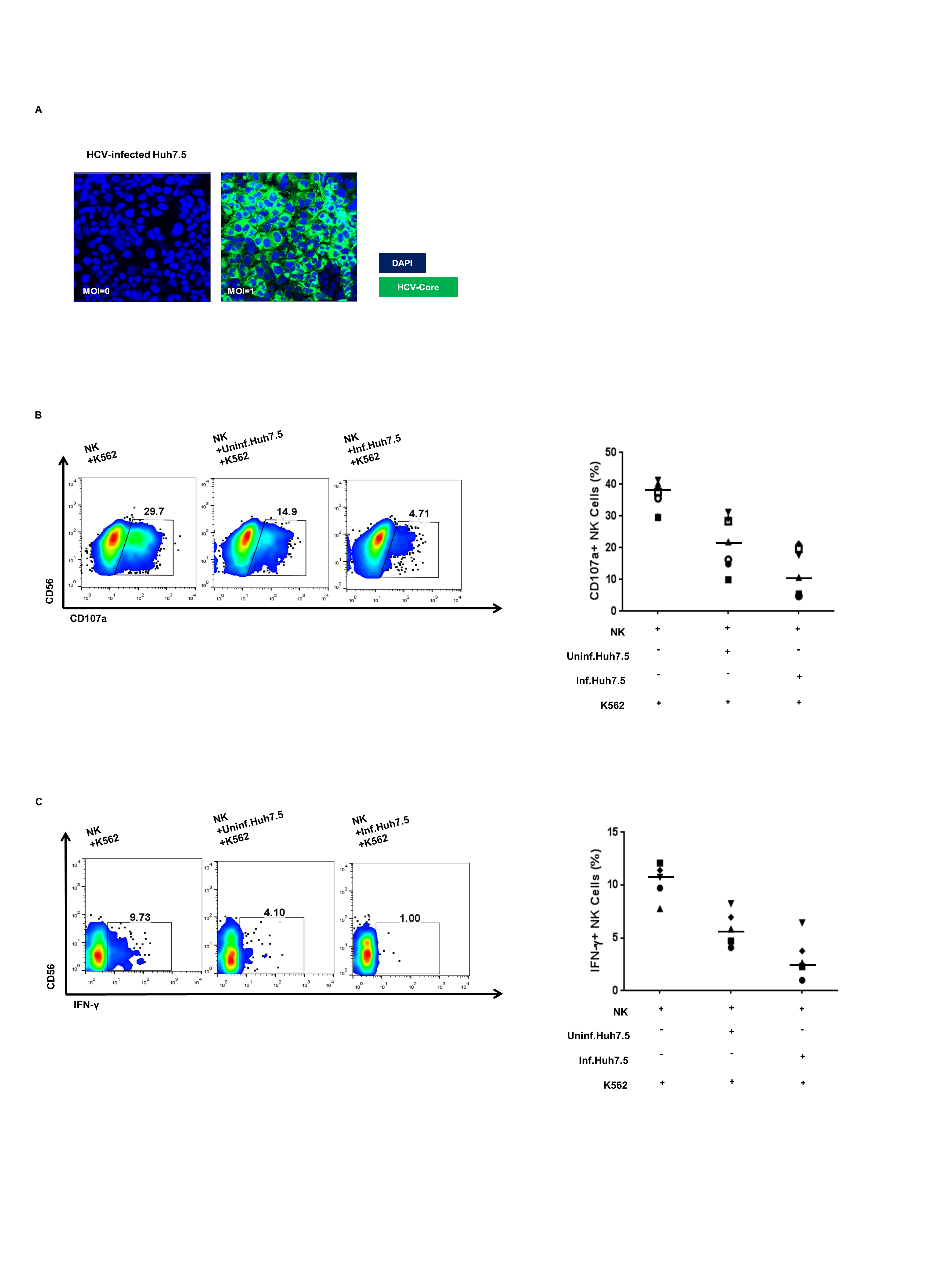

Supplement: S1 Fig — (A) Expression of HCV-Core protein in HCV-infected Huh-7.5 cells. Huh-7.5 cells were infected with HCV-JFH1 (MOI = 1), and three days later, cells were fixed and stained for HCV Core protein (green) immunofluorescence with DAPI (blue) nuclear staining. (B) NK cell degranulation after co-cultivation with HCV-infected Huh-7.5 cells was measured as described in Fig 1. (C) IFN-γ production by NK cells after co-cultivation with HCV-infected Huh-7.5 cells. IFN-γ production was assessed by intracellular staining of IFN-γ as described in Fig 1. (B) Representative pseudo color plots obtained for seven independent individuals. (C) Representative pseudo color plots obtained for five independent individuals. Bar presents the median value. (TIF) [file pone.0175793.s001.TIF]

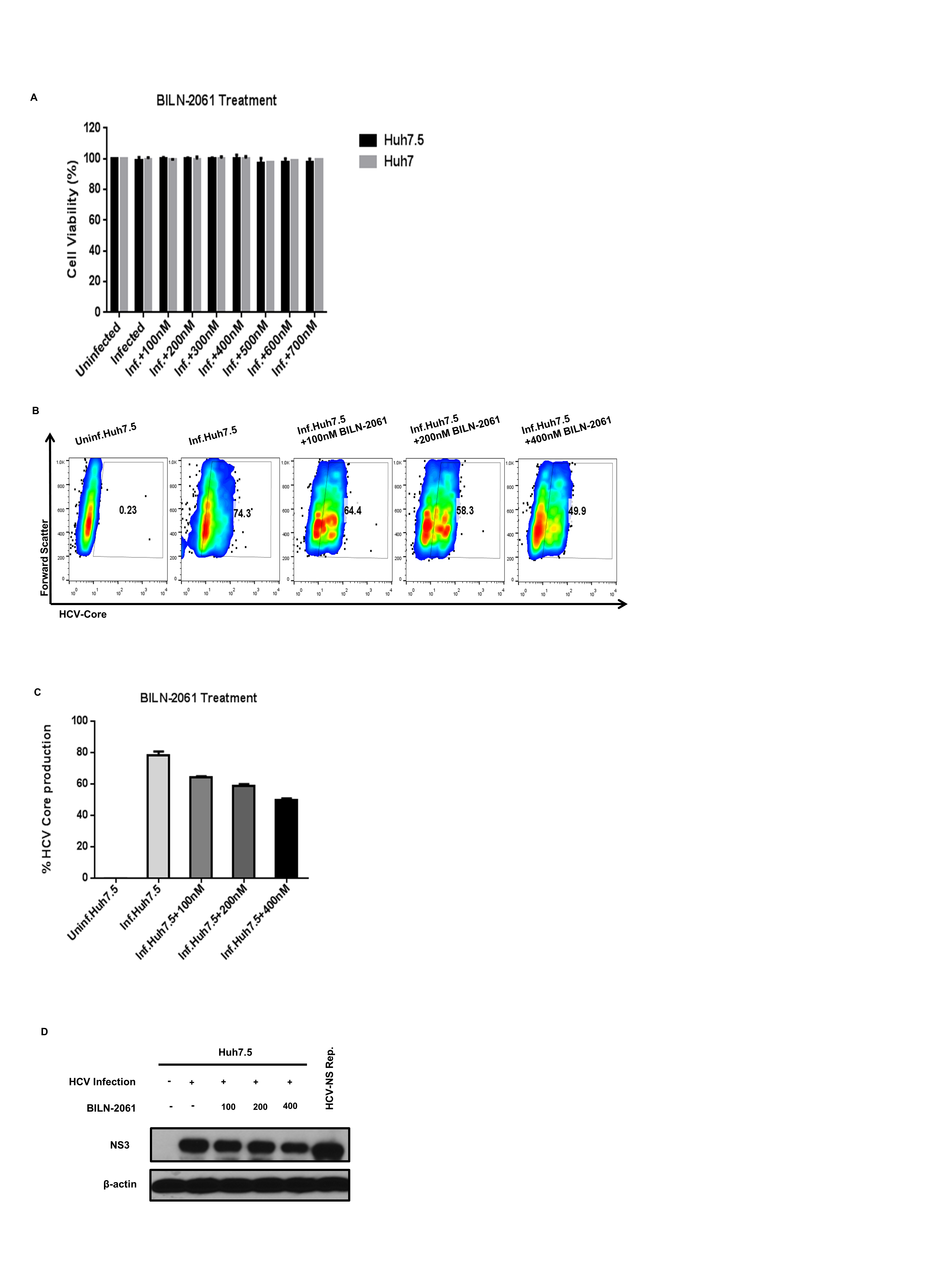

Supplement: S2 Fig — (A) Effect of BILN-2061 on cell viability. Huh-7.5 and Huh-7 cells were seeded in a 96-well flat bottom culture plate and infected with HCV at an MOI of 1 in Huh-7.5 cells and at an MOI of 10 in Huh-7 cells. After 4 h, the supernatant was removed, cells were transferred to complete DMEM, and treated with BILN-2061 (100–700 nM) for 48 h. Cell viability assay was performed using the CCK-8 kit (Dojindo Molecular Technologies, Japan). BILN-2061 did not affect cell viability up to 700 nM. (B-C) Effect of BILN-2061 on HCV replication. HCV-infected Huh-7.5 cells were treated with BILN-2061 (100–400 nM) for 48 h. HCV replication was determined by estimating HCV-Core expression levels by using flow cytometry (stained with anti-HCV-Core antibody). HCV replication in HCV-infected Huh-7.5 cells was reduced by BILN-2061-treatment in a dose-dependent manner. Representative pseudo color plots of the results from three independent experiments, and their bar graphs (C). (D) Effect of BILN-2061 on NS3 protein expression in HCV-infected Huh-7.5 cells. HCV-infected Huh-7.5 cells were treated with BILN-2061 (100–400 nM) for 48 h. Western blotting was performed using anti-HCV-NS3 and anti-β-actin antibodies. BILN-2061-treated HCV-infected Huh-7.5 cells reduced NS3 expression in a dose-dependent manner. Thus, the optimal concentration was determined to be 400 nM BILN-2061 for further experiments. (TIF) [file pone.0175793.s002.TIF]
